# Assessment and Comparison of N-Terminal-Probrain Natriuretic Peptide (NT-proBNP) in Saliva and Serum of Healthy Subjects, Periodontitis Patients, and Periodontitis Patients With Myocardial Infarction

**DOI:** 10.7759/cureus.67441

**Published:** 2024-08-21

**Authors:** Shraddha H Mohite, Girish Suragimath, Siddhartha Varma, Sameer A Zope, Ashwinirani SR

**Affiliations:** 1 Periodontology, School of Dental Sciences, Krishna Vishwa Vidyapeeth (Deemed to be University), Karad, IND; 2 Oral Medicine and Radiology, School of Dental Sciences, Krishna Vishwa Vidyapeeth (Deemed to be University), Karad, IND

**Keywords:** periodontitis, nt-probnp, myocardial infarction, cardiovascular diseases, biomarker

## Abstract

Introduction

Periodontitis is a multifactorial oral disease causing destruction of the periodontium. Systemic diseases can exacerbate periodontal inflammation through immune dysregulation. N-terminal-probrain natriuretic peptide (NT-proBNP) a prohormone, released by myocardial cells is a known biomarker for cardiovascular disease (CVD). Existing literature discloses a bidirectional relationship between periodontitis and CVD. NT-proBNP release can be regulated by mediators of the systemic inflammation. Cardiocyte NT-proBNP release might get stimulated through proinflammatory cytokines. NT-proBNP levels can also be influenced by systemic inflammation in the absence of cardiac dysfunction. Accordingly, we postulated that the inflammation of periodontium could aid in increased levels of NT-proBNP in serum and saliva in participants without cardiovascular disorders. Saliva is said to be the mirror of the body. Assessing NT-pro BNP in saliva allows for a non-invasive method. The present research evaluated the salivary and serum concentrations of NT-proBNP in a healthy group, patients suffering from periodontal disease and periodontal disease along with myocardial infarction (MI).

Material and method

A total of 90 patients, 30 in each group i.e., healthy group, periodontitis patients and patients suffering from periodontitis with myocardial infarction, were enrolled. The periodontitis patients were selected according to the Classification of Periodontal and Peri-implant Diseases and Conditions 2017. Patients clinically diagnosed with MI by the physician were selected following World Health Organization criteria for detection of MI. Case history was recorded and periodontal parameter analysis like plaque index (PI), gingival index (GI), probing pocket depth (PPD) and clinical attachment loss (CAL) were measured. Salivary and serum samples were collected from the participants after obtaining informed consent. The samples were subjected to human NT-proBNP sandwich type enzyme-linked immunosorbent assay (ELISA) for quantitative evaluation. The obtained data was analysed and compared using ANOVA, Tukey’s post hoc test and Pearson’s correlation. The p-value<0.05 was considered statistically significant.

Result

PI and GI were highest in subjects with periodontitis only (p<0.05). Patients suffering from periodontitis with MI exhibited significantly higher PPD and CAL values (p<0.05). Salivary and serum concentrations of NT-proBNP were significantly higher with p-value=0.000 in subjects suffering from periodontitis with MI. The salivary NT-proBNP levels were significantly higher than serum NT-proBNP levels in periodontitis and periodontitis with MI patients. The levels of NT-proBNP in periodontitis along with MI patients were 1.570 pg/mL in serum and 1.694 pg/mL in saliva.

Conclusion

Salivary NT-proBNP levels were highest in subjects affected from periodontitis along with MI. Elevated salivary NT-proBNP levels can be due to systemic inflammation and cardiovascular stress linking periodontitis to MI. The positive correlation between periodontal parameters and NT-proBNP levels validates the biomarker's role in reflecting the extent of periodontal destruction and its association with cardiovascular stress. Salivary NT-proBNP can be used as a non-invasive diagnostic marker for diagnosing periodontitis and MI. Future research could explore targeted therapies for the shared inflammatory pathways between periodontitis and MI.

## Introduction

Periodontal diseases are inflammatory, long-term tissue-destructive diseases that compromise the health of the periodontium. The ongoing progression of periodontal disease weakens the periodontium and ultimately causes tooth loss. Periodontal diseases arise from the interaction between the host's immunity, external environmental factors, pathogenic microorganisms, and genetic influences [1].

Chronic periodontal diseases serve as a source of ongoing inflammation and may play a role in the development of other systemic diseases and conditions associated with inflammation. The potential link between oral disorders and systemic diseases has been a subject of long-standing research. Periodontitis is recognized as a potential risk factor for conditions such as cardiovascular disorders, respiratory illnesses, diabetes, peripheral artery diseases, and adverse pregnancy outcomes like preterm and low birth weight babies [2].

Certain systemic disorders worsen both the frequency and severity of periodontitis by altering the host's immune response. Literature indicates an interdependent association between periodontitis and systemic illnesses and a mutual impact on the progression and outcomes of both conditions. The potential pathways connecting oral diseases with systemic conditions include the spread of infection from the oral cavity through transient bacteremia, systemic injury caused by circulating oral microbial toxins, and systemic inflammatory conditions instigated by the immune response to oral microorganisms.

The N-terminal prohormone of brain natriuretic peptide (NT-proBNP) is a precursor prohormone molecule containing a 76 amino acid inactive N-terminal protein segment, which is cleaved to release the active hormone, brain natriuretic peptide 32 (BNP). In response to increased volume and pressure, myocardial cells release pro-B-type natriuretic peptide (proBNP). This precursor is then processed to produce both the active BNP and the inactive N-terminal fragment known as NT-proBNP [3]. BNP has a half-life of about 20 minutes, while NT-proBNP remains in circulation for approximately 60-90 minutes [4]. The prolonged half-life of NT-proBNP facilitates substantial transfer from the bloodstream to saliva [5]. Elevated NT-proBNP levels result from inflammatory stimuli and may function as a potential biomarker in cardiovascular disease (CVD) owing to its extended half-life [6,7]. The release of NT-proBNP can be influenced by mediators of systemic inflammation. Systemic inflammation from periodontitis can elevate NT-proBNP levels due to the chronic inflammatory response that affects cardiovascular health. Cardiocyte NT-proBNP secretion may be triggered by proinflammatory cytokines such as interleukin-1β, interleukin-6, and tumor necrosis factor-alpha (TNF-α), and it can also be affected by systemic inflammation even in the absence of cardiac dysfunction [8,9]. We hypothesized that inflammation of the periodontium could contribute to elevated levels of NT-proBNP in both blood and saliva among individuals with and without cardiovascular disorders. Comparing NT-proBNP levels in saliva and serum across different patient groups is crucial because it can reveal how oral health relates to systemic conditions like cardiovascular disease. If salivary NT-proBNP levels correlate well with serum levels, saliva could be used as a non-invasive screening tool in dental check-ups to detect cardiovascular risk early. While existing literature extensively explores NT-proBNP’s role in myocardial infarction (MI) and periodontitis separately, our study addressed NT-proBNP’s relationship with periodontitis and MI. To the best of our knowledge this is the first study of its kind where we have explored NT-proBNP’s role in patients with periodontitis and MI. Hence, this study aimed to assess and compare NT-proBNP levels in serum and saliva among healthy individuals, patients with periodontitis, and periodontitis with MI, exploring the bi-directional role of NT-proBNP in periodontitis and MI.

## Materials and methods

Study design

This cross-sectional study was conducted in a single session at the Department of Periodontology, School of Dental Sciences, Karad, after receiving approval from the Ethical Committee of Krishna Vishwa Vidyapeeth (Deemed to be University), Karad (Protocol number: 014/2022-2023, dated: 09/08/2022).

Sample size estimation

Ninety subjects attending the outpatient section underwent a thorough periodontal assessment. The sample size for the study was arrived at by power analysis. The power analysis was established by G*Power version 3.0.1 (Franz Faul Universitat, Kiel, Germany). A minimum calculated sample size of 87, rounded up to 90 samples (30 per group across three groups), provided 80% power to detect significant differences, with an effect size of 0.34 and a significance level of 0.05.

Inclusion and exclusion criteria

Patients providing consent for participation in the study were selected. The criteria for assessing MI and periodontitis were carefully defined and verified using established diagnostic standards. Patients clinically pre-diagnosed with MI by the physician were selected following World Health Organization criteria. They were selected based on: 1. Clinical history of ischaemic type chest pain lasting for more than 20 minutes; 2. Changes in serial ECG tracings; 3. Rise and fall of serum cardiac biomarkers such as creatinine kinase MB fraction and troponin [10]. Generalized stage II grade B periodontitis patients were chosen according to Classification of Periodontal and Peri-implant Diseases and Conditions 2017 [11]. Stage II Grade B periodontitis patients were selected to provide a clear representation of moderate periodontitis with a balanced progression rate, making them ideal for studying disease characteristics and minimizing variability in severity and disease progression. Patients with systemic conditions apart from MI, pregnant and lactating females, patients with periodontal therapy within past three months and patients with history of tobacco use were left out of the study. They were excluded to reduce confounding variables that could influence the progression and response of periodontitis. Systemic conditions like diabetes can significantly impact periodontal health and affect the results. Tobacco use is a well-known risk factor that exacerbates periodontal disease and could introduce bias in the study. Similarly, recent periodontal therapy could alter the natural disease state, making it difficult to accurately assess the results.

Selection of subjects

Patients were informed about the study objectives, and those meeting the inclusion and exclusion criteria were enrolled after receiving informed consent. A stratified random sampling method was used to reduce the selection bias. Basic demographic information, socioeconomic status, and lifestyle details were recorded using a customized data collection proforma and were subjected to periodontal examination. All the observations and recordings were done by a single trained examiner to ensure consistency.

Grouping of the subjects

Participants were categorized into the following groups: Group A (n=30) included periodontally healthy subjects; Group B (n=30) comprised patients with Stage II Grade B periodontitis; Group C (n=30) consisted of patients with Stage II Grade B periodontitis who also had MI. All participants underwent clinical evaluation for Plaque Index (PI) and Gingival Index (GI). Probing pocket depth (PPD) and Clinical Attachment Level (CAL) were measured using a Williams's graduated periodontal probe (Hu-Friedy, Rotterdam, Netherlands).

Sample collection

Two ml of unstimulated whole saliva was collected by following circadian rhythm, between 10 am and 12 pm, two hours after the last meal. The collection time was chosen based on the circadian rhythm to minimize hormonal fluctuations and other physiological variations that could affect the parameters being measured. By standardizing the collection time, the study aimed to reduce variability and ensure that all samples were comparable. To prevent contamination, participants were asked to rinse their mouths thoroughly with distilled water before collection. They were instructed to refrain from talking and asked to accumulate saliva in their mouth before expectorating it into a collection vessel until the desired volume was collected. The saliva samples were then centrifuged at 1000 rpm at 2-8°C for 15 minutes to remove any cell debris. Approximately 0.5 ml of the supernatant was transferred into labeled 1.5 ml Eppendorf tubes. Each tube was labeled with a unique tracking identifier number. The samples were stored at -80°C until analysis.

Serum samples were collected under strict aseptic conditions. Two ml of blood was drawn into a plain vacutainer. The blood samples were then allowed to clot at room temperature. After one hour, the serum was separated by centrifuging the blood at 1000 rpm for 20 minutes. The isolated serum of 0.5 ml was promptly transferred into an Eppendorf tube and stored at -80°C, with each tube labeled with a unique tracking identifier number.

Estimation of serum salivary NT-proBNP levels

Serum and salivary NT-ProBNP concentrations were measured using a sandwich enzyme-linked immunosorbent assay kit (Human NT-ProBNP ELISA Kit; ELK Biotechnology Co., Ltd., Wuhan, China). The sensitivity of this ELISA kit is 15 pg/mL and the detection range is 39.07- 2500 pg/mL. The assay followed the manufacturer's instructions and levels of NT-proBNP were recorded using an ELISA Reader. NT-proBNP levels were calculated using a quadratic regression equation, and the resulting data were compiled and organized in an Excel spreadsheet (version 2019; Microsoft, Redmond, WA, USA).

Statistical analysis

**Figure 1:**
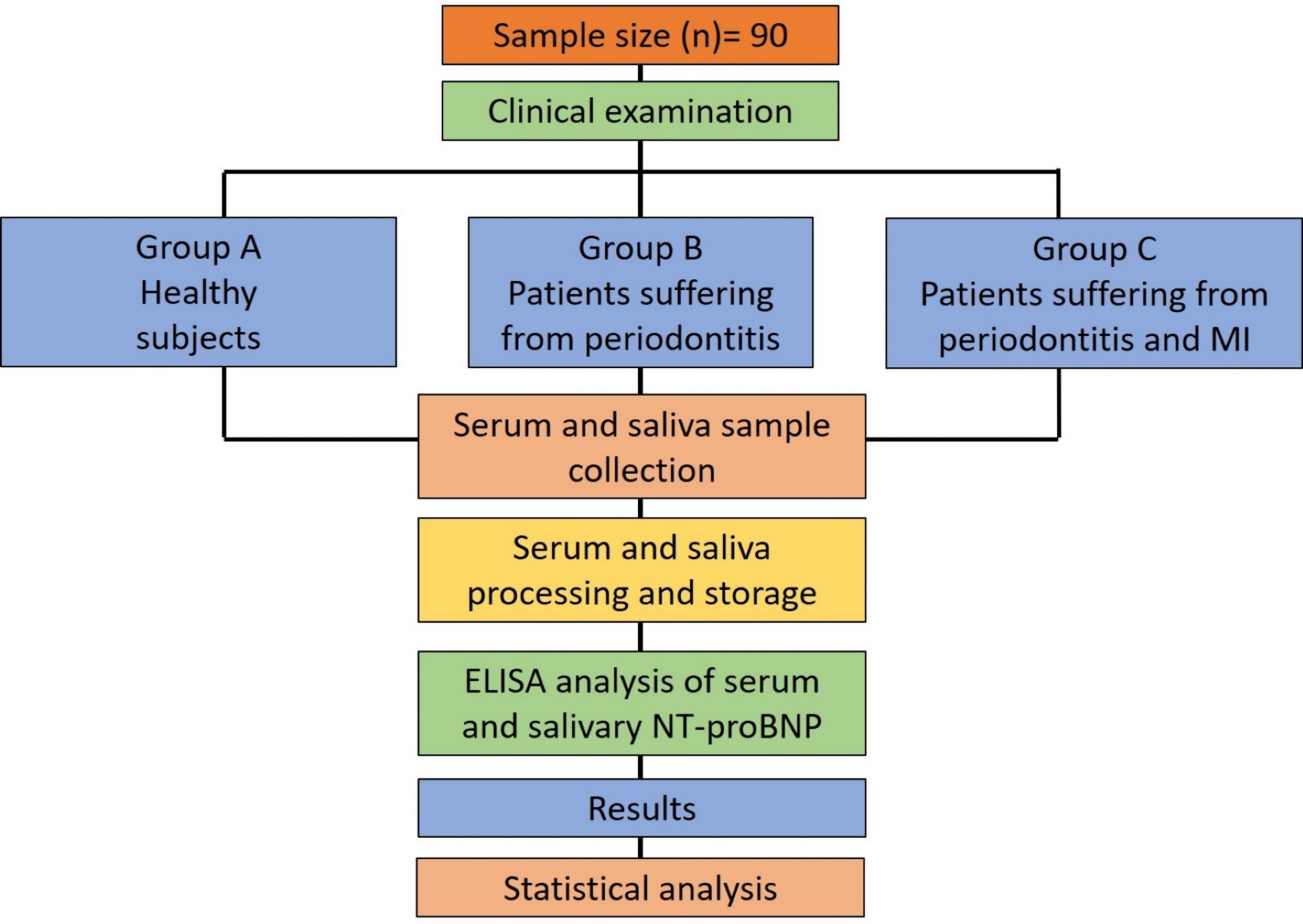
Flow diagram of the methodology. ELISA: enzyme-linked immunosorbent assay, NT-proBNP: N-terminal-probrain natriuretic peptide

All statistical analyses were performed using IBM SPSS statistical software (SPSS version 25; IBM, Armonk, NY, USA). One-way ANOVA test was used to acquire mean PI, GI, PPD, CAL, serum and salivary NT-proBNP levels. A pairwise comparison was carried out using Tukey’s post hoc t-test. To test whether there is a significant relationship between these parameters, the Pearson correlation coefficient was derived. The steps of the methodology are explained in Figure 1.

## Results

**Figure 2:**
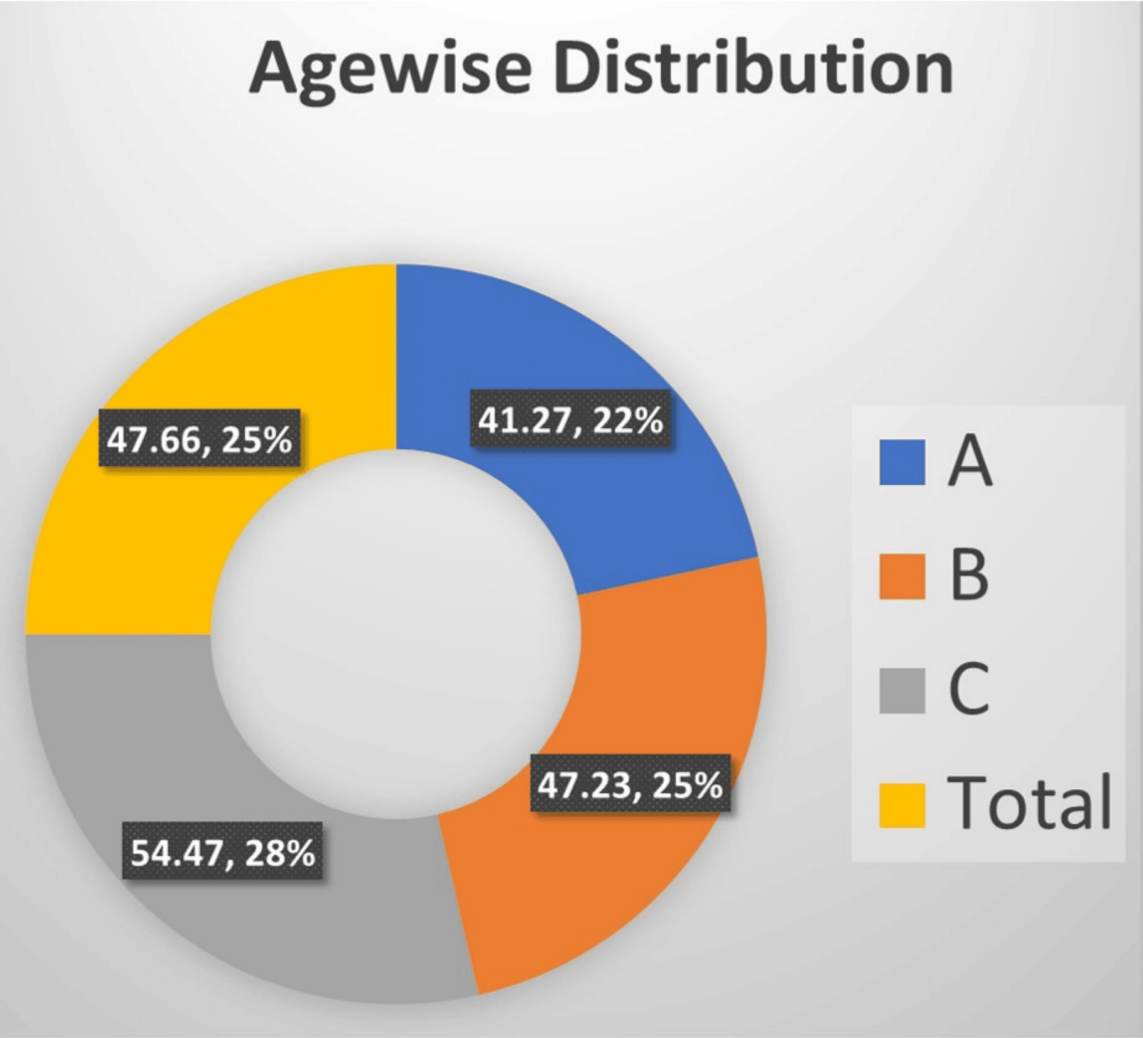
Age distribution of the patients. Group A: Healthy subjects; Group B: Patients suffering from periodontitis only; Group C: Patients suffering from periodontitis and myocardial infarction.

The study included 90 patients aged 30-65 years, with 62 males (68.88%) and 28 females (31.11%). The overall mean age was 47.66±11.02 years. The mean age of participants for Groups A, B, and C were 41.27 years, 47.23 years, and 54.47 years, respectively (Figure 2).

PI and GI were highest in group B with means of 2.183±0.581 and 5.533±0.860 respectively. PPD and CAL were observed more in group C with means of 7.030±1.351 mm and 9.830±1.206 mm respectively. A statistically significant difference was exhibited between all three groups for all periodontal parameters (p-value=0.000) (Table 1).

**Table 1:** Intergroup periodontal parameters comparison. ¶p-value<0.05: statistically significant. Group A: Healthy subjects; Group B: Patients suffering from periodontitis only; Group C: Patients suffering from periodontitis and myocardial infarction. PI: Plaque index; GI: Gingival index; PPD: Probing pocket depth; CAL: Clinical attachment level; mm: millimeters; p-value: probability value.

Parameter	Group	Mean	Standard Deviation	Standard Error	95% Confidence Interval for Mean		Minimum	Maximum	p-value
					Lower Bound	Upper Bound			
PI	A	0.090	0.119	0.022	0.046	0.134	00.0	00.4	0.000^¶^
	B	2.183	0.581	0.106	1.966	2.400	01.7	03.4	
	C	2.147	0.488	0.089	1.964	2.329	01.6	03.4	
GI	A	0.077	0.094	0.017	0.042	0.112	00.0	00.3	0.000^¶^
	B	5.533	0.860	0.157	5.212	5.855	04.0	06.0	
	C	5.200	0.997	0.182	4.828	5.572	04.0	06.0	
PPD (mm)	A	0.000	0.000	0.000	0.000	0.000	00.0	00.0	0.000^¶^
	B	6.170	0.913	0.167	5.830	6.510	05.0	08.0	
	C	7.030	1.351	0.247	6.530	7.540	05.0	09.0	
CAL (mm)	A	0.000	0.000	0.000	0.000	0.000	00.0	00.0	0.000^¶^
	B	8.630	1.098	0.200	8.220	9.040	07.0	10.0	
	C	9.830	1.206	0.220	9.380	10.280	08.0	12.0	

The pairwise comparison using Tukey’s post hoc test showed a statistically significant difference in PI and GI values between Group A to Group B, and C (p-value=0.000). No statistically significant differences were seen between Group B and C. Statistically significant differences were seen for PPD and CAL values between Group A to Group B and C, as well as between Group B and C (Table 2).

**Table 2:** Pairwise comparison of periodontal parameters using Tukey’s post hoc test. ¶p-value<0.05: statistically significant, ¥p>0.05: statistically not significant. Group A: Healthy subjects; Group B: Patients suffering from periodontitis only; Group C: Patients suffering from periodontitis and myocardial infarction. PI: Plaque index; GI: Gingival index; PPD: Probing pocket depth; CAL: Clinical attachment level; mm: millimeters; p-value: probability value.

Dependent Variable	Group	Group	Mean Difference	Standard Error	95% Confidence Interval		p-value
					Lower Bound	Upper Bound	
PI	A	B	-2.093	0.115	-2.366	-1.820	0.000^¶^
		C	-2.057	0.115	-2.330	-1.784	0.000^¶^
	B	A	2.093	0.115	1.820	2.366	0.000^¶^
		C	0.037	0.115	-0.236	0.310	0.945^¥^
	C	A	2.057	0.115	1.784	2.330	0.000^¶^
		B	-0.037	0.115	-0.310	0.236	0.945^¥^
GI	A	B	-5.457	0.197	-5.926	-4.988	0.000^¶^
		C	-5.123	0.197	-5.592	-4.654	0.000^¶^
	B	A	5.457	0.197	4.988	5.926	0.000^¶^
		C	0.333	0.197	-0.136	0.802	0.213^¥^
	C	A	5.123	0.197	4.654	5.592	0.000^¶^
		B	-0.333	0.197	-0.802	0.136	0.213^¥^
PPD (mm)	A	B	-6.167	0.243	-6.750	-5.590	0.000^¶^
		C	-7.033	0.243	-7.610	-6.450	0.000^¶^
	B	A	6.167	0.243	5.590	6.750	0.000^¶^
		C	-0.867	0.243	-1.450	-0.290	0.002^¶^
	C	A	7.033	0.243	6.450	7.610	0.000^¶^
		B	0.867	0.243	0.290	1.450	0.002^¶^
CAL (mm)	A	B	-8.633	0.243	-9.210	-8.050	0.000^¶^
		C	-9.833	0.243	-10.410	-9.250	0.000^¶^
	B	A	8.633	0.243	8.050	9.210	0.000^¶^
		C	-1.200	0.243	-1.780	-0.620	0.000^¶^
	C	A	9.833	0.243	9.250	10.410	0.000^¶^
		B	1.200	0.243	0.620	1.780	0.000^¶^

A statistically significant difference was exhibited between all three groups for NT-proBNP serum and salivary levels (p-value=0.000). Both serum and saliva levels of NT-proBNP were significantly higher in Group C followed by Group B and least in Group A (Table 3).

**Table 3:** Intergroup comparison of NT-proBNP serum and salivary levels. ¶p-value<0.05: statistically significant. Group A: Healthy subjects, Group B; Patients suffering from periodontitis only; Group C: Patients suffering from periodontitis and myocardial infarction. pg/mL: Picograms per millilitre; p-value: probability value, NT-proBNP: N-terminal-probrain natriuretic peptide

Parameter	Group	Mean	Standard Deviation	Standard Error	95% Confidence Interval for Mean		Minimum	Maximum	p-value
					Lower Bound	Upper Bound			
NT-pro BNP: Serum (pg/ml)	A	0.219	0.088	0.016	0.186	0.252	0.113	0.569	0.000^¶^
	B	0.997	0.215	0.039	0.917	1.078	0.603	1.501	
	C	1.570	0.459	0.083	1.399	1.742	0.613	2.315	
NT-pro BNP: Saliva (pg/ml)	A	0.230	0.148	0.027	0.175	0.285	0.144	0.882	0.000^¶^
	B	1.189	0.353	0.064	1.057	1.321	0.556	1.818	
	C	1.694	0.448	0.081	1.527	1.862	0.807	2.541	

The pairwise comparison using Tukey’s post hoc test revealed a statistically significant difference in NT-proBNP serum and salivary levels between all three groups, Group A to Group B and C, as well as between Group B to C (p-value=0.000) (Table 4).

**Table 4:** Pairwise comparison of salivary and serum NT-proBNP levels using Tukey’s post hoc test. ¶p-value<0.05: statistically significant. Group A: Healthy subjects; Group B: Patients suffering from periodontitis only; Group C: Patients suffering from periodontitis and myocardial infarction. pg/mL: picograms per millilitre; p-value: probability value, NT-proBNP: N-terminal-probrain natriuretic peptide

Dependent Variable	Group	Group	Mean Difference	Standard Error	95% Confidence Interval		p-value
					Lower Bound	Upper Bound	
NT-pro BNP: Serum (pg/ml)	A	B	-0.778	0.076	-0.961	-0.595	0.000^¶^
		C	-1.351	0.076	-1.534	-1.168	0.000^¶^
	B	A	0.778	0.076	0.595	0.961	0.000^¶^
		C	-0.573	0.076	-0.756	-0.389	0.000^¶^
	C	A	1.351	0.076	1.168	1.534	0.000^¶^
		B	0.573	0.076	0.389	0.756	0.000^¶^
NT-pro BNP: Saliva (pg/ml)	A	B	-0.959	0.087	-1.169	-0.749	0.000^¶^
		C	-1.464	0.087	-1.674	-1.254	0.000^¶^
	B	A	0.959	0.087	0.749	1.169	0.000^¶^
		C	-0.505	0.087	-0.714	-0.295	0.000^¶^
	C	A	1.464	0.087	1.254	1.674	0.000^¶^
		B	0.505	0.087	0.295	0.714	0.000^¶^

Periodontal parameters like PI, GI, PPD and CAL were correlated with NT-proBNP levels in serum and saliva using Pearson's correlation. All the variables exhibited a strong positive correlation among all periodontal parameters (PI, GI, PPD, CAL) and NT-proBNP levels in both serum and saliva. This signifies that with an increase in periodontal parameters there is a concomitant increase in salivary and serum NT-proBNP levels. This implies that patients suffering from periodontitis are at an increased risk of MI (Table 5).

**Table 5:** The Pearson's correlation among the different variables. ** Correlation is significant at the 0.01 level. PI: Plaque index; GI: Gingival index; PPD: Probing pocket depth; CAL: Clinical attachment level; mm: millimetre; pg/mL: picograms per millilitre, NT-proBNP: N-terminal-probrain natriuretic peptide

Parameters		PI	GI	PPD	CAL	NT-proBNP: Serum	NT-proBNP: Saliva
PI	Pearson Correlation	1	0.894^**^	0.873^**^	0.900^**^	0.709^**^	0.756^**^
GI	Pearson Correlation	0.894^**^	1	0.891^**^	0.915^**^	0.734^**^	0.750^**^
PPD (mm)	Pearson Correlation	0.873^**^	0.891^**^	1	0.968^**^	0.805^**^	0.814^**^
CAL (mm)	Pearson Correlation	0.900^**^	0.915^**^	0.968^**^	1	0.808^**^	0.829^**^
NT-proBNP: Serum (pg/ml)	Pearson Correlation	0.709^**^	0.734^**^	0.805^**^	0.808^**^	1	0.956^**^
NT-proBNP: Saliva (pg/ml)	Pearson Correlation	0.756^**^	0.750^**^	0.814^**^	0.829^**^	0.956^**^	1

## Discussion

Periodontal diseases are a chronic inflammatory infectious disease of the supporting tissues of the teeth, that causes periodontal attachment loss, and bone loss. Periodontitis occurs in response to a variety of agents both of host and bacterial origin [12]. Microorganisms responsible for periodontal diseases trigger the release of endotoxins and inflammatory cytokines into the bloodstream. This creates a biological burden initiating inflammatory, atherogenic, and thromboembolic events. Inflammatory activation has been observed in patients with congestive heart failure (CHF) regardless of the underlying cause. This suggests a potential association between CVD and periodontal diseases [13].

Natriuretic peptides are vital in maintaining cardiovascular balance and extracellular fluid levels. These peptides exert vasodilatory, diuretic, and natriuretic effects, causing an increase in intravascular volume [14]. The interrelationship between chronic periodontitis and concentration of NT-proBNP in saliva, serum, and GCF has been investigated in a considerable number of researches [15]. But to the best of our knowledge, this is one of the first studies that has assessed and compared the concentration of NT-proBNP in serum and saliva of periodontitis patients and patients suffering from periodontitis along with myocardial infarction.

Our study found that male patients were affected more by MI than female patients. A study conducted in the Indian population by Srivastava et al. has demonstrated equivalent results to our research, where out of 305 patients diagnosed with MI, 274 were males and 30 were females [16]. Contradictory results were observed in a study by Gupta et al., who showed equal distribution of MI in males and females [17]. This inconsistency may be attributed to the uneven gender distribution in our study.

In the present study, the mean age of periodontitis patients was 47.23±10.76 years. Goulart et al. showed similar results where periodontitis patients exhibited a mean age of 48 years [18]. This indicates that periodontitis predominantly affects middle-aged and older individuals.

In our study, the mean value of PI and GI was observed more in Group B followed by Group C, and least in Group A. This was similar to the study done by Sreenivasan et al. [19]. The increased plaque accumulation and elevated gingival index in periodontitis are primarily due to the maturation of microbial biofilms, which harbor pathogenic bacteria that increase inflammation.

In the present research, the mean PPD and CAL were highest in patients suffering from periodontitis and MI and lowest in healthy subjects. A study by Khosravi et al. showed similar results where PPD and CAL increase with the increase in periodontal destruction severity [20]. This denotes that the risk for MI is significantly higher in individuals with periodontitis and vice versa. Elevated NT-proBNP levels in conjunction with poor periodontal parameters suggest that periodontal disease may contribute to systemic inflammation, which can exacerbate cardiovascular conditions.

In our study, the levels of serum NT-proBNP were higher among patients with periodontitis in comparison to the healthy group. This is in agreement with the study conducted by Leira et al., where the results showed the levels of serum NT-proBNP higher in subjects with periodontitis compared to the control group [15]. Similarly, according to Isola et al. and Fazal et al., the levels of NT-proBNP increased with periodontal inflammation [21,1]. Proinflammatory cytokines like IL-1β, IL-6, and TNF-α cause inflammation stimulating cardiocyte expression of natriuretic peptides. This can be due to the hemodynamic stress occurring during inflammation. Inflammation affects the hemodynamic load resulting in higher NT-proBNP levels in serum.

In our study, the analysis of salivary NT-proBNP levels among all three groups revealed a statistically significant difference. This is in agreement with Leira et al. and Sosnin et al., where the levels of salivary NT-proBNP were elevated in patients with periodontitis compared to the control group, suggesting that NT-proBNP levels in saliva rise with increasing periodontal inflammation [15,22].

In our study, we observed that the levels of salivary NT-proBNP exceeded serum NT-proBNP levels. Similar findings were observed in a study carried out by Foo et al., where they reported that salivary NT-proBNP was 100% specific in identifying patients with heart failure when compared with healthy controls [23].

The current research found a strong positive correlation between salivary and serum NT-proBNP levels and the indices of PI, GI, PPD, and CAL. Our study is in conjunction with Provan et al. and Leira et al., where they discovered the periodontal parameters destruction increased with elevated levels of NT-proBNP [9,15]. Our findings suggested that NT-proBNP levels rose concurrently with the extent of periodontal destruction. It offers valuable insights into the potential involvement of NT-proBNP in the advancement of periodontal disease.

In the present study, we observed that NT-proBNP levels increased in correlation with an increase in periodontal disease parameters. Highest levels were observed in patients suffering from periodontitis with MI. These findings highlight the potential role of periodontal disease as a contributing factor for MI. Periodontitis can trigger systemic events such as atherogenesis and thrombogenesis through the release of endotoxins and inflammatory cytokines into the bloodstream. Furthermore, periodontal pathogens and their byproducts can enter the bloodstream, directly affecting the cardiovascular system and potentially leading to increased NT-proBNP levels. Therefore, NT-proBNP could be a promising biomarker for investigating this association [24].

The current research highlights the utilization of saliva for NT-proBNP biomarker analysis in patients suffering from periodontitis with MI. The study done by Bellagambi et al. is in favor with our research [25]. NT-proBNP is formed in response to increased cardiac wall stress and is released into the bloodstream. Whole saliva is an "ultra-filtrate" of blood due to its reflection of biological activity and health status. NT-proBNP can be excreted via saliva through various mechanisms, including ultrafiltration from blood plasma, passive diffusion, and active transport via salivary gland cells. Unlike blood, saliva samples can be collected easily and non-invasively reducing psychological stress, making it ideal for critical subjects such as the elderly and individuals with disabilities.

Limitations

This study's limitations include a small sample size, which may hinder result extrapolation. The examiner was not blinded to prevent observer bias. Longitudinal studies at multiple points are needed to check for the fluctuations. Additionally, the study focused on specific populations or demographics, potentially restricting the generalizability of the findings. Further research involving multiple clinical settings and diverse racial and population groups is necessary to validate and extend the applicability of these results.

## Conclusions

Patients suffering from both periodontitis and periodontitis along with myocardial infarction exhibited a strong positive correlation between serum and salivary NT-proBNP levels. The substantial periodontal degradation, together with elevated NT-proBNP levels, highlights a significant interplay between oral and cardiovascular markers. Unravelling these correlations improves diagnostic accuracy and therapeutic approaches for managing patients with concurrent periodontal and cardiovascular diseases.

The positive associations observed between NT-proBNP and all measured periodontal parameters reinforce the bidirectional relationship between periodontitis and MI. This suggests that each condition may worsen the progression of the other. The salivary NT-proBNP levels were significantly higher than serum NT-proBNP levels. While salivary testing offers advantages such as ease of collection and patient comfort, it emerges as a promising non-invasive tool for diagnosing both periodontitis and MI. This highlights its potential in clinical practice for systemic health management. The interaction between periodontal and cardiovascular markers could transform treatment strategies, emphasizing the need for an integrated approach to managing patients with both conditions. Incorporating regular periodontal evaluations and targeted treatments into cardiovascular care could potentially lower the risk of myocardial infarction by effectively managing periodontal inflammation.

For future perspective, longitudinal interventional studies with a large sample size should be conducted to enhance the outcome. The correlation of serum and salivary NT-proBNP with other cardiovascular biomarkers found in periodontal diseases should be conducted to compare and correlate further. Periodontitis is a treatable disease, further studies should be performed to assess the potential impact of periodontal therapy on the concentration of this molecule, which can serve as a surrogate marker of cardiovascular diseases.
